# Supplementary figures and images for: Diverse functions of homologous actin isoforms are defined by their nucleotide, rather than their amino acid sequence
Source: eLife. 2017 Dec 15;6:e31661. doi: 10.7554/eLife.31661 (PMC5794254; doi:10.7554/eLife.31661)

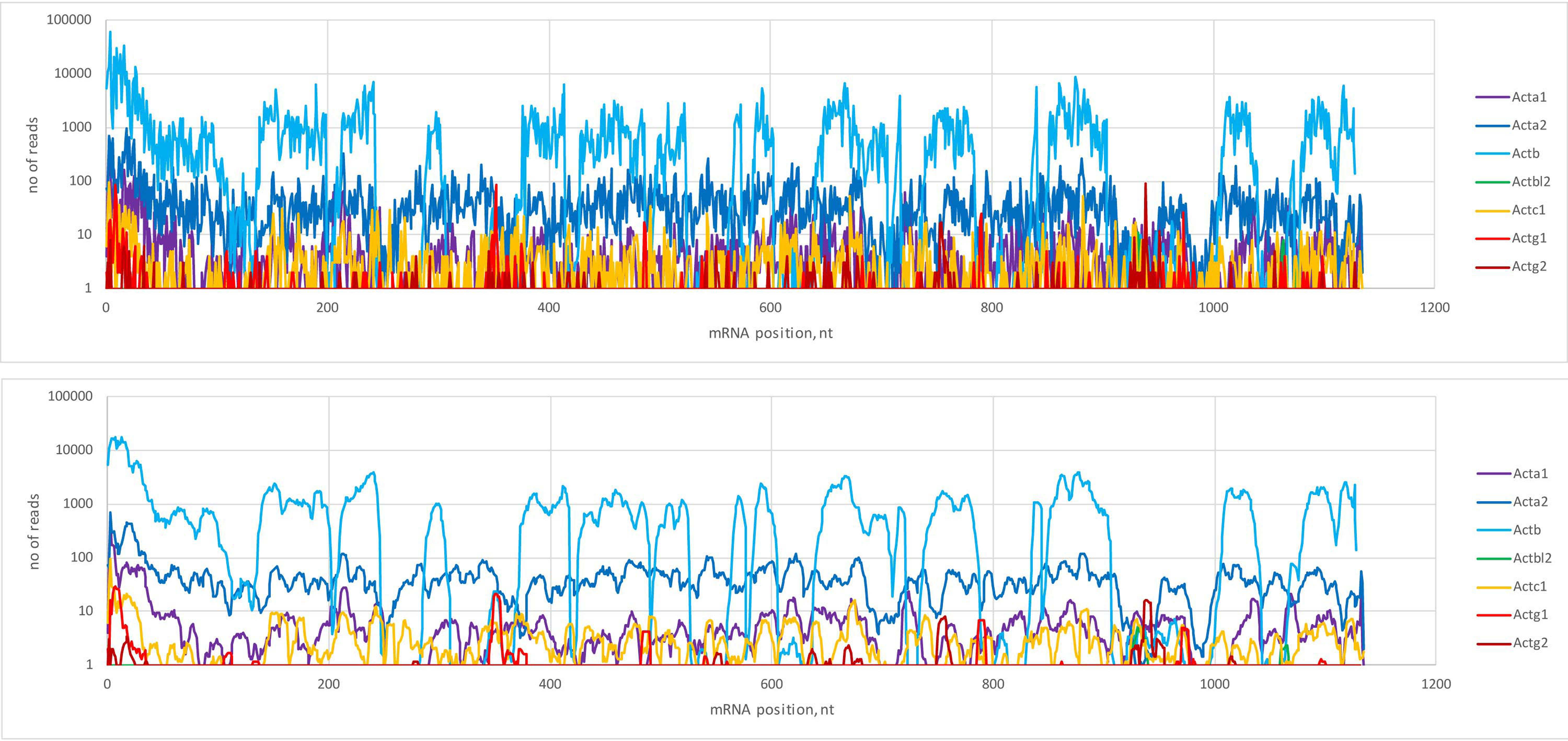

Supplement: Table 1—source data 1. — Bottom panel shows the coarse curves for the data on top. [file elife-31661-table1-data1.jpg]

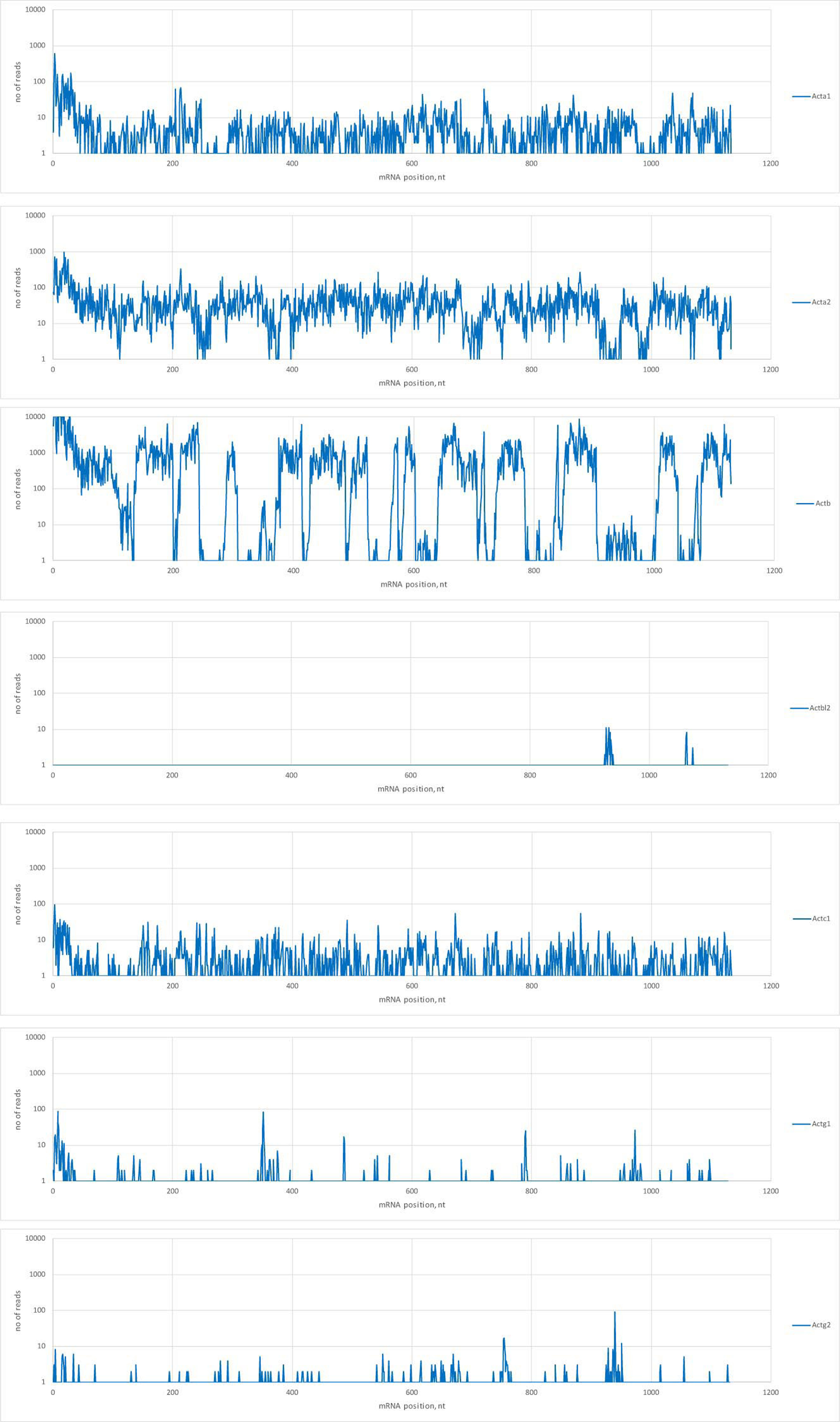

Supplement: Table 1—source data 2. [file elife-31661-table1-data2.jpg]

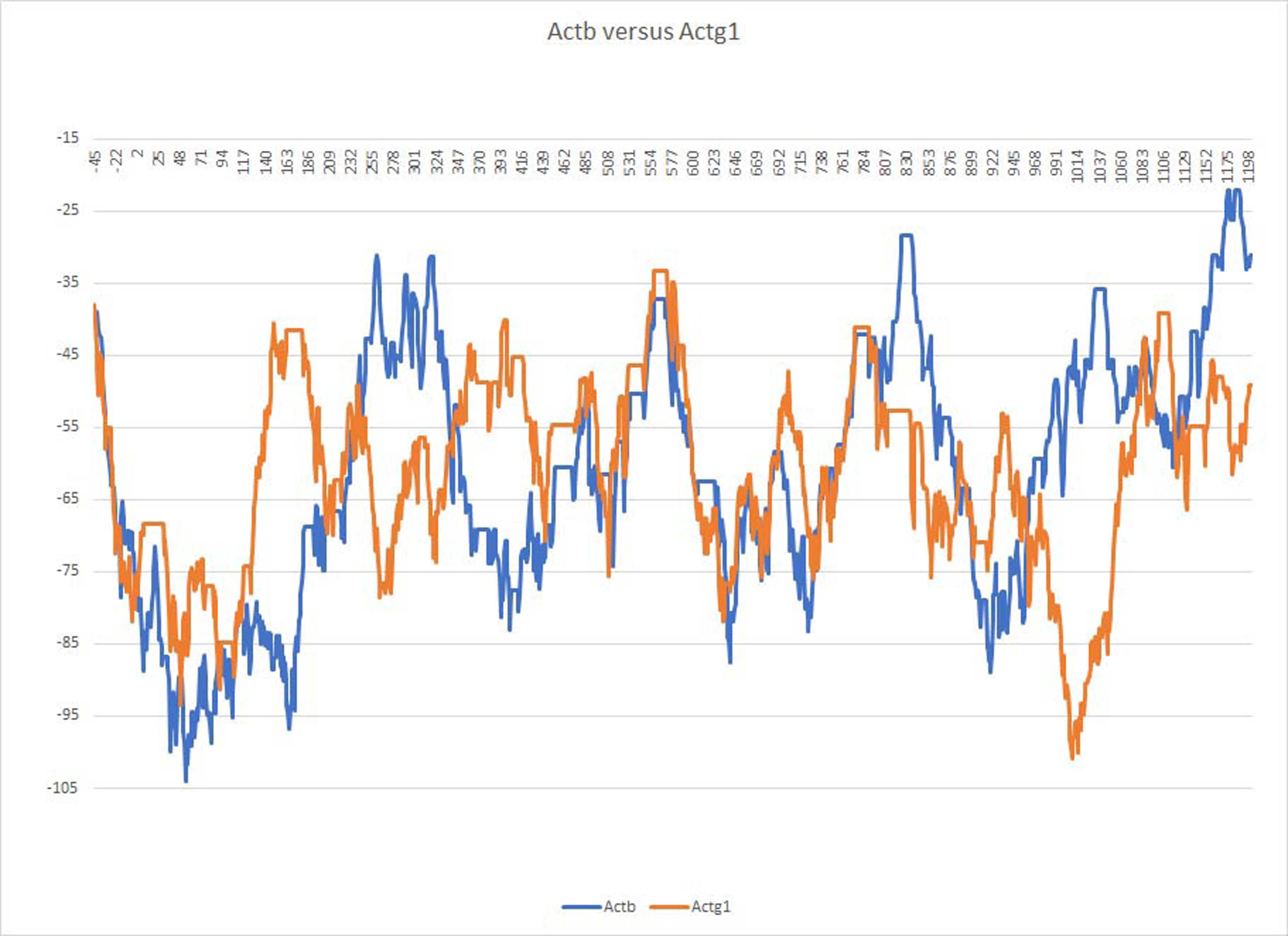

Supplement: Table 1—source data 3. — Plot shows distance in nucleotides (x axis, 0 indicates the first ATG of the coding sequence) versus free energy (y axis). [file elife-31661-table1-data3.jpg]

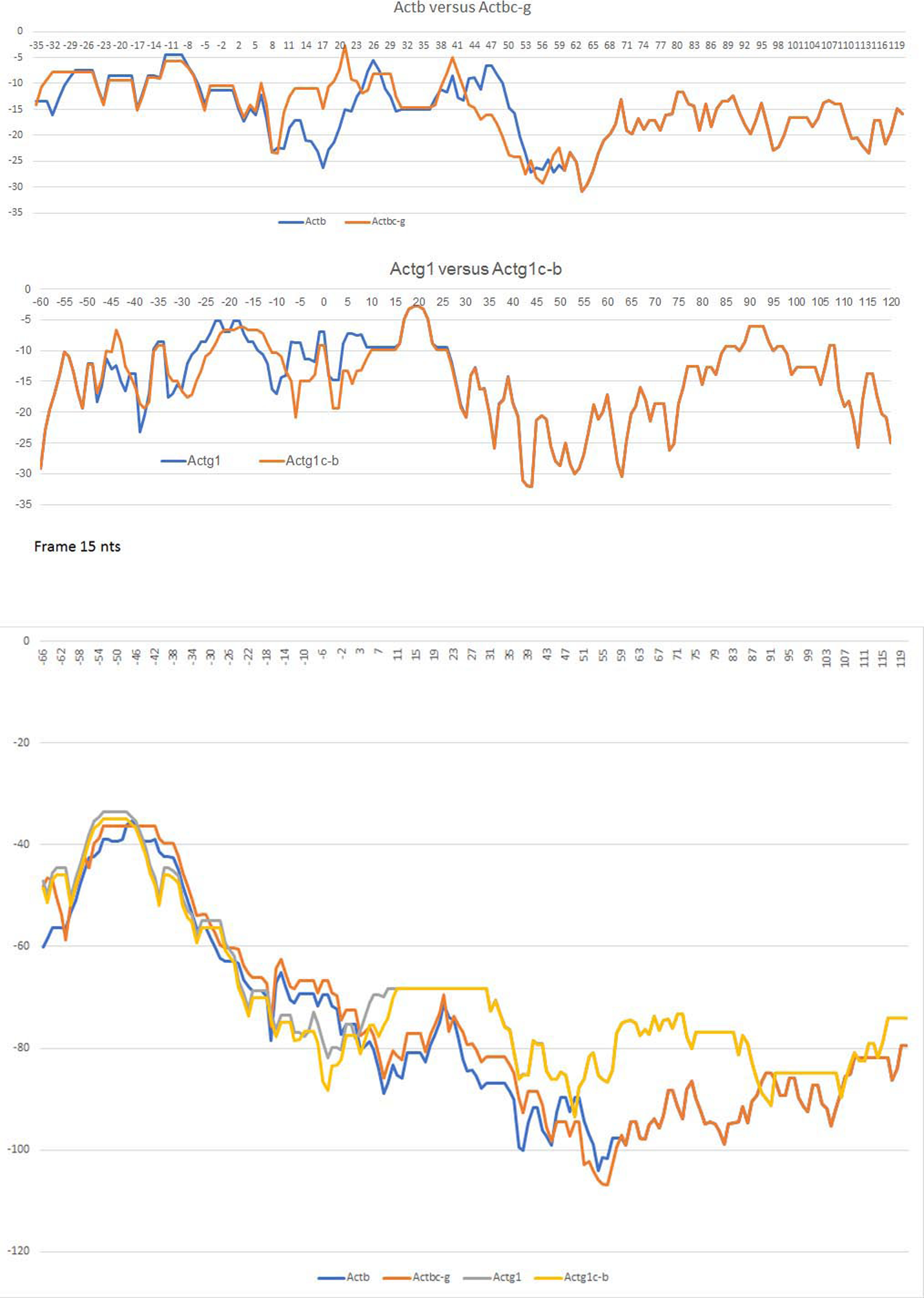

Supplement: Table 1—source data 4 — Plot shows distance in nucleotides (x axis, 0 indicates the first ATG of the coding sequence) versus free energy (y axis). β-coded γ− actin mRNA is predicted to have a more relaxed structure around the translation initiation site, while being indistinguishable throughout the rest of the sequence. [file elife-31661-table1-data4.jpg]
